# Migration in large femoral head ceramic-on-ceramic monoblock acetabular component compared with modular ceramic-on-polyethylene acetabular component in total hip arthroplasty using radiostereometric analysis with 5 years’ follow-up: a randomized controlled trial

**DOI:** 10.2340/17453674.2026.46138

**Published:** 2026-07-01

**Authors:** Caner IÇLI, Brechtje HESSELING, Jantsje H PASMA, Ian BLOM, Rolf M BLOEM, Nina M C MATHIJSSEN

**Affiliations:** 1 Reinier Haga Orthopaedic Centre, Zoetermeer; 2 Department of Orthopaedic Surgery, Reinier de Graaf Gasthuis, Delft; 3 Department of Radiology, Reinier de Graaf Gasthuis, Delft, the Netherlands

## Abstract

**Background and purpose:**

An uncemented ceramic-on-ceramic (CoC) monoblock cup is an acetabular component designed for younger and more active patients. We compared migration, revision rates, and clinical results of this uncemented CoC monoblock acetabular component with a commonly used uncemented ceramic-on-polyethylene (CoPE) modular cup.

**Methods:**

In this prospective randomized controlled trial we used radiostereometric analysis (RSA) to study primary THAs that were performed after being allocated 1:1 to uncemented CoC monoblock acetabular component or to uncemented ceramic-on-polyethylene (CoPE) modular cup. Radiographs were obtained directly postoperatively, and at 1.5, 3, 6, 12, 24, and 60 months postoperatively. Migration of both cups was calculated using model-based RSA (mbRSA) in terms of translations and rotations. Revision rates and patient-reported outcome measures (PROMs) (Harris Hip Score, Oxford Hip Score, EQ-5D-5L) were also registered.

**Results:**

We included 50 patients. The monoblock cup shows a statistically significant higher total translation compared with the modular cup (mean difference –0.71 mm, 95% confidence interval 0.10–1.31) at 5 years’ follow-up. Both cups show an increased translation along the x- and y-axes over time, which stabilized after 3 months. No statistically significant differences were found in the rotation patterns of the cups. However, both cups show an increased rotation about the z-axis over time, which decreased between 2 and 5 years’ follow-up. All PROMs improved after surgery and remained stable.

**Conclusion:**

Although the CoC monoblock cup showed a higher translation than the CoPE modular cup, the revision rates and clinical outcomes in both groups were similar. The CoC monoblock cup showed acceptable mid-term outcomes comparable with the CoPE modular cup.

Currently, the most used uncemented acetabular cup system for total hip arthroplasty (THA) in The Netherlands is a bispherical modular cup system [1]. The modular cup used with ceramic on highly cross-linked polyethylene (CoPE) has shown high long-term survival rates and great clinical improvement [2-7]. Another uncemented large-head ceramic-on-ceramic (CoC) monoblock cup system with a hemispherical head is designed for younger and more active patients to improve survival and stability and to provide a greater range of motion before impingement [8-10]. While mid-term results show high survival rates and good functional results, evidence on long-term results for this monoblock cup system is limited [11-13]. Both cup types have been compared in the past and showed no differences in risk of revision and loosening [14,15]. However, functional outcomes seem to be better in THA with CoC cup systems than THA with CoPE cup systems [16].

Given the lack of long-term results for the monoblock cup system, a valuable method to enhance our understanding of the cup’s performance is radiostereometric analysis (RSA) [17]. With RSA, translational and rotational migration of arthroplasty components can be measured accurately. Previous studies showed that migration values over time can predict revision risk due to aseptic loosening [18,19]. Such migration patterns have not yet been studied for these cup systems.

Therefore, our primary aim was to investigate early fixation and migration patterns of the uncemented large-head CoC monoblock cup system in THA using model-based RSA during 5 years of follow-up and to compare these results with the results of the uncemented CoPE modular cup system combined with a polyethylene liner. The secondary aim was to evaluate clinical outcomes during 5 years of follow-up of both cups. We hypothesized that the migration patterns of the uncemented large-head CoC monoblock cup are equal to the uncemented CoPE modular cup system.

## Methods

### Design and participants

This was a single-center randomized controlled trial, with all included patients undergoing THA between May 2015 and January 2019 at the Reinier de Graaf hospital (Delft, the Netherlands). Patients were included if they were 18–75 years old with a body mass index (BMI) < 35, had a diagnosis of primary or secondary osteoarthritis or avascular necrosis of the hip joint and an American Society of Anesthesiologists Physical Status classification (ASA) of grade 1–3. Patients were excluded if they had undergone THA in the contralateral hip less than 6 months before the current surgery or had unsatisfactory outcomes (Harris Hip Score < 85) after contralateral THA more than 6 months ago. Patients were also excluded if they underwent a major surgical procedure in the 12 weeks prior to the current surgery, were diagnosed with a myocardial infarct or cerebrovascular accident within 3 months prior to the current surgery, were mentally disabled, had an active infection or current malignancy, uncontrolled hypertension, or a known history of alcohol or drug abuse.

During inclusion, patients were randomized using pre-prepared sealed, opaque envelopes to receive either the uncemented large head CoC monoblock Maxera cup or the uncemented CoPE modular Allofit cup, both combined with the uncemented M/L Taper Femoral stem, all produced by Zimmer Biomet, Indiana, USA. The opaque envelopes were shuffled and then numbered 1–50. At the moment of inclusion, the envelope was opened in front of the patient.

The study is reported according to CONSORT guidelines.

### Surgery and implants

All operations were performed by 1 orthopedic surgeon, specialized in hip surgery (RMB), using the straight lateral approach. During surgery, 5–9 tantalum markers with a diameter of 1.0 mm were inserted into the reamed acetabulum, before implanting the allocated cup. Because the Allofit cup is a modular system, a highly cross-linked polyethylene liner (Durasul Alpha insert, Zimmer Biomet, IN, USA) was inserted. In both groups the THA was completed by implantation of the uncemented M/L Taper Femoral stem with a ceramic femoral head (Biolox Delta, Zimmer Biomet, IN, USA), consisting of 75% aluminum oxide and 25% zirconia. Postoperatively, full weightbearing was allowed.

### Outcomes

#### Radiostereometric analysis

During this study, we followed the RSA guidelines as noted in Kaptein et al. and the International Organization for Standardization (ISO) [20]. To follow up the migration rates of the acetabular cup, RSA and conventional radiographs were obtained directly postoperatively (after weightbearing, on the day of surgery or first postoperative day) and 1.5, 3, 6, 12, 24, and 60 months postoperatively. RSA radiographs were performed using a standard RSA set-up consisting of 2 roentgen tubes (1 fixed and 1 portable tube) (DigitalDiagnost C90 and the MobileDiagnost wDR [Philips, Best, the Netherlands]). The tubes were positioned at an angle of 40° to each other and a distance of 1.2 m to the table. Participants were positioned in a supine position. A calibration cage (Medis, Leiden, the Netherlands) was positioned posteriorly to the concerned hip, under the table. The axis from the left to the right was parallel to the x-axis, the longitudinal axis of the leg was parallel to the y-axis, and the antero-posterior axis was parallel to the z-axis. The images were digitally saved in DICOM format at a resolution of 6.8 pixels/mm and a 16-bit grey-scale resolution.

Migration was computed using model-based RSA software (Version 4.2; RSAcore, LUMC, Leiden, the Netherlands) and computer-aided design (CAD) models of the cup. First, for each time point the 3-dimensional position of the cup (the migrating object) relatively to the center of gravity of the tantalum markers in the pelvic bone (the reference object) was calculated. A consistent set of markers throughout the follow-up radiographs was used. To ensure that the rotation measurements were usable, a minimum of 3 bone markers should have been visible over the follow-up time points and the markers had to be stable (mean error < 0.35 mm). In case of less than 3 visible and stable bone markers, a condition number of > 120, and/or a rigid body fitting error of > 0.35 mm, the measurement was excluded from further analysis. To include RSA radiographs with less than 3 markers or high condition numbers, mean marker models (MC model) were used [21].

RSA analysis was performed by 2 researchers with extensive training and experience in RSA (IB and RO). To calculate the migration, the RSA radiographs were compared with the reference RSA radiograph taken directly postoperatively. Due to the symmetrical design of the cups, the local y-axis was fixed to ignore the rotations about the local y-axis. Migrations were expressed in the global coordinate system and divided in translations (T, in mm) along and rotations (R, in degrees) about the medial (x), cranial (y), and anterior (z) axes. The origin of the coordinate system was located in the center of gravity of the cup. Besides the translations and rotations in 3 directions, also the total translation (TT = Euclidean sum of x, y, and z migration) and total rotation (TR = Euclidean sum of x, y, and z rotation) were calculated.

At 1 year postoperatively, a double examination was performed to calculate the precision. The precision was defined as the standard deviation (SD) and the bias as the mean of the migration between the 2 examinations with the first examination as the reference. The precision interval (PI) was defined as 1.96 x SD and represents the expected clinical precision. Measured migrations within the PI were deemed inconclusive, as they might be measurement errors.

#### Patient-reported outcome measures (PROMs)

To investigate the clinical outcomes, the Harris Hip Score (HHS), Oxford Hip Score (OHS), and the EQ-5D-5L were measured at baseline, 1.5, 3, 6, 12, 24, and 60 months. The HHS consists of pain- and function-related questions and clinical outcome measures such as deformity and range of motion, with a score ranging from 0 (worst outcome) to 100 (best outcome) [22]. The OHS measures pain intensity and functional limitations using 12 items, with a total score ranging from 0 (worst outcome) to 48 (best outcome) [23]. The EQ-5D-5L is a quality of life questionnaire using 5 domains (mobility, self-care, activities in daily living, pain, and anxiety) with a scoring of 1 (best outcome) to 5 points (worst outcome) on each domain. It also contains a visual analogue score (VAS) from 0 (worst outcome) to 100 (best outcome) on how healthy the patient feels [24].

#### Complications

Complication rates were also assessed and defined as any perioperative or postoperative hip-related complications defined as dislocation, fracture, revision for any reason, collapse, persistent pain, and death.

### Statistics

Patient characteristics and baseline PROMs are presented in means and SD, or in case of non-normality by median and interquartile range (IQR). For categorical variables numbers are presented.

Linear mixed models were used to analyze migration results to determine the main effect of time and the cup on migration and their interaction, considering the longitudinal nature and missing values. Estimated means and mean differences are presented with corresponding 95% confidence interval (CI).

We tested differences in proportions of responders and non-responders with chi-square tests or, in case of cell counts < 5, Fisher’s exact tests, to compare the PROMs between the 2 groups at 2 and 5 years’ follow-up. Differences are presented by odds ratio (OR) with CI.

To define responders and non-responders to the treatment, we used thresholds of the PROMs score as described in the literature. For the HHS, Singh et al. defined the minimal clinically important improvement (MCII) to be 18 points and 15.9 points at 2 and 5 years, respectively. The threshold for moderate improvement was 39.6 and 40.1 points change at 2 and 5 years, respectively [25]. For the OHS, Beard et al. describe a minimal clinically important difference (MCID) of 7.5 points [23].

The level of significance for all tests was set at P < 0.05. Missing data was not imputed.

All data was analyzed using SPSS version 28.0.1.0 (IBM Corp Armonk, NY, USA).

### Sample size calculation

The clinically relevant difference in migration between the 2 groups was set at 0.2 mm. With a significance level of 5%, 90% power, and an estimated SD of 0.15 mm, we would need 13 patients in each group. To account for loss to follow-up (e.g., dropout or technical issues of placing the beads or obtaining RSA radiographs), we aimed for 25 patients in each group.

### Ethics, registration, funding, data sharing, and disclosures

The study was approved by our regional Medical Ethics Committee (METC Leiden, Den Haag, Delft (METC-LDD) in The Netherlands, METC-no. Z21.014). The study is registered in the Dutch trial register OMON (Overzicht van medisch-wetenschappelijk onderzoek in Nederland, NL-OMON53013) [26]. All included patients signed the study-specific informed consent form prior to the study. This study is funded by Zimmer Biomet. Data cannot be shared. To perform clinical trials, the research department receives grants from Zimmer Biomet and Stryker. Zimmer Biomet was not involved in design, conduct, analysis, and writing of this study.

Complete disclosure of interest forms according to ICMJE are available on the article page, doi: 10.2340/17453674.2026.46138

## Results

**Figure 1:**
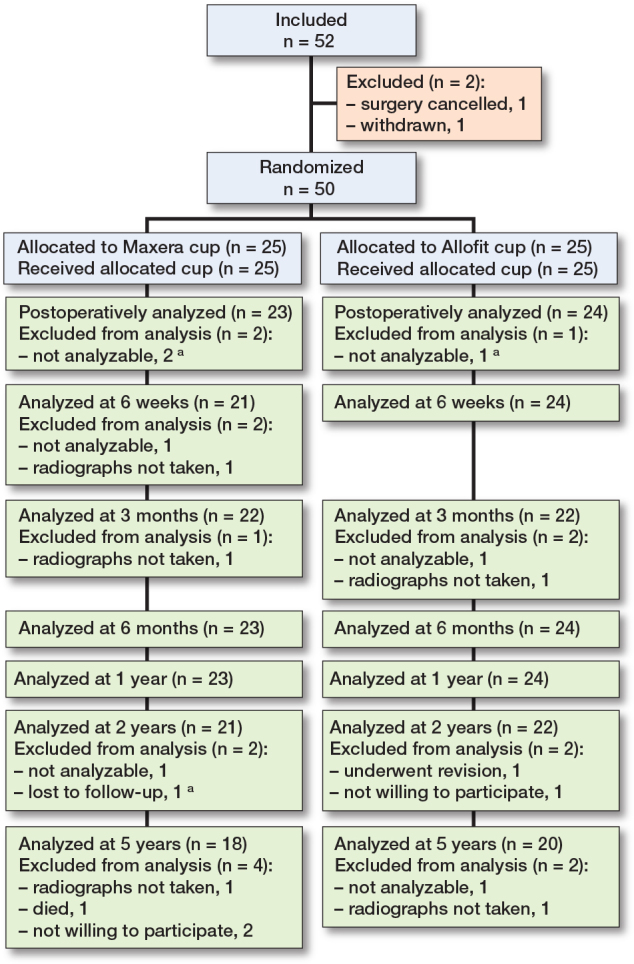
RSA follow-up flowchart of the ceramic-on-ceramic monoblock Maxera cup and the ceramic-on-polyethylene modular Allofit cup. **^a^** Excluded from every follow-up moment after being mentioned.

After assessing eligibility, 52 patients were included in this study. After inclusion, 2 patients were excluded due to cancelled surgery and withdrawal. 25 patients were allocated to each group. After surgery, we lost a few patients to follow up due to various reasons (Figure 1). Patient characteristics and the baseline PROMs are shown in Table 1.

**Table 1:** Patient demographics at baseline. Values are mean (standard deviation) unless otherwise specified

Item	CoC MB	CoPE mod
	cup (n = 25)	cup (n = 25)
Age, years	63 (8.7)	61 (8.3)
Female sex, n	15	14
Operated on left side, n	12	14
Height, m	1.72 (0.1)	1.74 (0.1)
Weight, kg	88.2 (15.1)	82.6 (15.9)
Body mass index	29.8 (3.6)	27.1 (3.4)
Anesthesia, n		
Spinal	23	23
General anesthesia	2	1
Combined general anesthesia and spinal	0	1
Surgery time, min	101 (16)	91 (16)
Peroperative blood loss, mL	488 (255)	376 (167)
Preoperative diagnosis, n		
Coxarthrosis	24	25
Avascular necrosis of femoral head	1	0
ASA class, n		
1	7	12
2	14	13
3	4	0
Harris Hip Score	60 (16)	58 (11)
Oxford Hip Score	26 (9.0)	23 (7.0)
EQ5D-5L domains, median (IQR)		
Mobility	3 (3–4)	3 (3–4)
Self-care	1 (1–3)	2 (1–3)
ADL	3 (2–4)	3 (2–4)
Pain	3 (3–4)	3 (3–4)
Anxiety	1 (1–2)	1 (1–1)
EQ5D-5L VAS Health score	69 (18)	70 (18)
Cup sizes, mm, median (IQR)	52 (3)	52 (8)
Femoral head sizes, mm, median (IQR)	40 (4)	32 (0)

ADL = activities of daily living; ASA = American Society of Anesthesiologists Physical Status. CoC MB = ceramic-on-ceramic monoblock. CoPE mod = ceramic-on-polyethylene modular.

### Radiostereometric analysis

**Figure 2:**
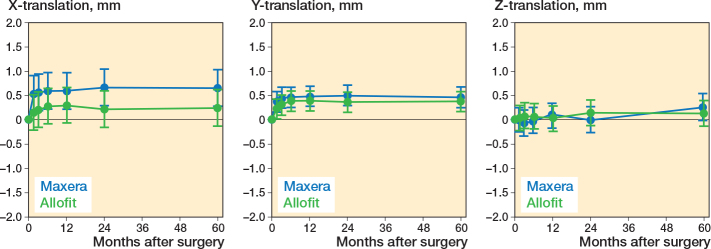
Predicted translation of the ceramic-on-ceramic monoblock Maxera cup and the ceramic-on-polyethylene modular Allofit cup during 5 years’ follow-up presented by estimated mean and 95% confidence interval.

**Figure 3:**
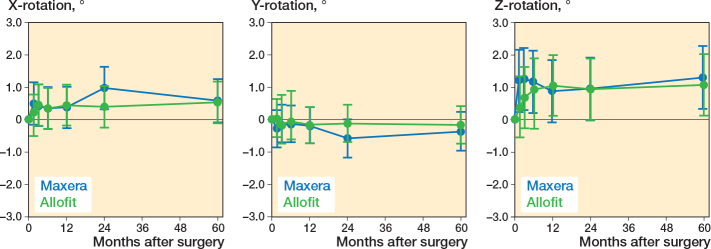
Predicted rotation of the ceramic-on-ceramic monoblock Maxera cup and the ceramic-on-polyethylene modular Allofit cup during 5 years’ follow-up presented by estimated mean and 95% confidence interval.

**Figure 4:**
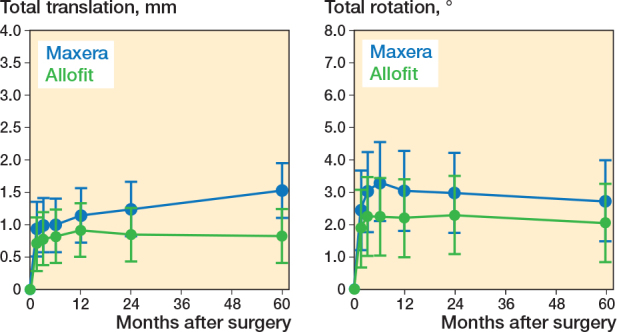
Predicted total translation and rotation of the ceramic-on-ceramic monoblock Maxera cup and the ceramic-on-polyethylene modular Allofit cup during 5 years’ follow-up presented by estimated mean 95% confidence interval.

We analyzed the descriptive statistics of the RSA analysis, including the number of markers used, the condition number, and the mean error of rigid-body fitting (Table 2). In 8 monoblock CoC cups and 5 modular CoPE cups a mean marker model was used. Table 3 shows the precision intervals obtained with the double examination performed at 1 year after surgery. Double examinations were obtained from 18 patients in each group. Migration of both cups seems to occur in the first 6 weeks with stabilization between 6 weeks and 3 months postoperatively (Figures 2–4 and Tables 4–5).

**Table 2:** Descriptives of analysis of each follow-up point stratified by type of cup. Values are mean (standard deviation)

Time point	Number of markers ^a^		Condition number		Mean error of rigid body ^a^	
	CoC MB	CoPE mod	CoC MB	CoPE mod	CoC MB	CoPE mod
6 weeks	5.5 (1.7)	6.0 (1.6)	102.4 (56.8)	78.6 (28.2)	0.202 (0.063)	0.167 (0.072)
3 months	5.5 (1.7)	6.1 (1.6)	103.7 (54.3)	78.8 (27.1)	0.203 (0.075)	0.161 (0.060)
6 months	5.5 (1.7)	6.0 (1.6)	100.9 (54.7)	78.6 (28.2)	0.189 (0.076)	0.178 (0.067)
1 year	5.5 (1.7)	6.0 (1.6)	100.9 (54.7)	78.6 (28.2)	0.197 (0.080)	0.188 (0.067)
2 years	5.5 (1.7)	5.9 (1.7)	104.5 (55.5)	78.6 (28.0)	0.201 (0.072)	0.201 (0.067)
5 years	5.7 (1.8)	6.1 (1.7)	92.7 (35.1)	80.0 (27.7)	0.237 (0.075)	0.224 (0.073)

a Analyses using mean marker models are not included.

For abbreviations, see Table 1.

**Table 3:** Precision results of double examinations of the CoC monoblock and CoPE modular cup

	CoC monoblock (n = 18)		CoPE modular (n = 18)	
	Mean (SD)	PI	Mean (SD)	PI
Translation, mm				
x	0.14 (0.26)	0.51	–0.001 (0.15)	0.28
y	–0.03 (0.08)	0.15	–0.04 (0.09)	0.17
z	0.07 (0.19)	0.38	0.12 (0.34)	0.67
Total	0.30 (0.21)		0.28 (0.27)	
Rotation, degrees				
x	0.06 (0.58)	1.14	–0.07 (0.35)	0.69
y	–0.02 (0.65)	1.27	0.11 (0.38)	0.74
z	0.25 (0.55)	1.08	0.06 (0.49)	0.96
Total	0.89 (0.54)		0.54 (0.46)	

PI = precision interval

**Table 4:** Translation during 5 years’ follow-up stratified by type of cup. Values are count and estimated mean with 95% confidence interval (CI)

Item	n	CoC monoblock	n	CoPE modular	Mean difference (CI)
X-translation, mm					
6 weeks	21	0.53 (0.15 to 0.91)	24	0.15 (–0.22 to 0.51)	–0.38 (–0.91 to 0.15)
3 months	22	0.56 (0.18 to 0.94)	22	0.21 (–0.17 to 0.58)	–0.35 (–0.89 to 0.17)
6 months	23	0.59 (0.21 to 0.97)	24	0.27 (–0.10 to 0.64)	–0.32 (–0.85 to 0.21)
1 year	23	0.59 (0.22 to 0.97)	24	0.29 (–0.08 to 0.66)	–0.30 (–0.83 to 0.23)
2 years	21	0.66 (0.28 to 1.04)	22	0.21 (–0.16 to 0.58)	–0.45 (–0.98 to 0.08)
5 years	18	0.64 (0.26 to 1.03)	20	0.24 (–0.14 to 0.61)	–0.41 (–0.94 to 0.13)
Y-translation, mm					
6 weeks	21	0.37 (0.15 to 0.58)	24	0.22 (0.01 to 0.43)	–0.15 (–0.45 to 0.15)
3 months	22	0.45 (0.24 to 0.67)	22	0.29 (0.09 to 0.50)	–0.16 (–0.46 to 0.14)
6 months	23	0.46 (0.24 to 0.67)	24	0.38 (0.18 to 0.59)	–0.07 (–0.37 to 0.23)
1 year	23	0.48 (0.27 to 0.69)	24	0.39 (0.18 to 0.59)	–0.09 (–0.39 to 0.20)
2 years	21	0.50 (0.28 to 0.71)	22	0.36 (0.15 to 0.57)	–0.14 (–0.44 to 0.16)
5 years	18	0.46 (0.24 to 0.67)	20	0.37 (0.16 to 0.58)	–0.09 (–0.39 to 0.22)
Z-translation, mm					
6 weeks	21	0.01 (–0.26 to 0.28)	24	0.04 (–0.23 to 0.30)	0.03 (–0.35 to 0.41)
3 months	22	–0.07 (–0.34 to 0.20)	22	0.08 (–0.18 to 0.35)	0.15 (–0.23 to 0.53)
6 months	23	–0.02 (–0.29 to 0.25)	24	0.07 (–0.20 to 0.33)	0.09 (–0.29 to 0.46)
1 year	23	0.08 (–0.19 to 0.35)	24	0.02 (–0.25 to 0.28)	–0.06 (–0.44 to 0.32)
2 years	21	–0.01 (–0.28 to 0.27)	22	0.14 (–0.12 to 0.41)	0.15 (–0.23 to 0.53)
5 years	18	0.26 (–0.03 to 0.54)	20	0.13 (–0.14 to 0.40)	–0.13 (–0.52 to 0.26)
Total translation, mm					
6 weeks	21	0.94 (0.52 to 1.37)	24	0.71 (0.30 to 1.13)	–0.23 (–0.83 to 0.37)
3 months	22	1.01 (0.58 to 1.44)	22	0.80 (0.38 to 1.22)	–0.21 (–0.81 to 0.39)
6 months	23	1.01 (0.58 to 1.43)	24	0.84 (0.42 to 1.25)	–0.17 (–0.77 to 0.42)
1 year	23	1.16 (0.73 to 1.58)	24	0.94 (0.52 to 1.35)	–0.22 (–0.82 to 0.37)
2 years	21	1.25 (0.83 to 1.68)	22	0.86 (0.44 to 1.28)	–0.39 (–0.99 to 0.21)
5 years	18	1.54 (1.11 to 1.98)	20	0.84 (0.42 to 1.26)	–0.71 (–1.31 to –0.10)

**Table 5:** Rotation patterns over time. Values are count and estimated mean with 95% confidence interval (CI)

Item	n	CoC monoblock	n	CoPE modular	Mean difference (CI)
X-rotation, degrees					
6 weeks	21	0.50 (–0.15 to 1.16)	24	0.13 (–0.50 to 0.77)	–0.37 (–1.28 to 0.54)
3 months	22	0.45 (–0.20 to 1.10)	22	0.45 (–0.19 to 1.10)	0.00 (–0.91 to 0.92)
6 months	23	0.35 (–0.29 to 0.98)	24	0.35 (–0.30 to 1.00)	–0.01 (–0.91 to 0.90)
1 year	23	0.39 (–0.26 to 1.03)	24	0.46 (–0.18 to 1.09)	0.07 (–0.83 to 0.98)
2 years	21	0.99 (0.33 to 1.65)	22	0.40 (–0.25 to 1.04)	–0.59 (–1.51 to 0.33)
5 years	18	0.59 (–0.08 to 1.26)	20	0.53 (–0.12 to 1.18)	–0.07 (–1.00 to 0.87)
Y-rotation, degrees					
6 weeks	21	–0.28 (–0.86 to 0.31)	24	0.03 (–0.54 to 0.59)	0.30 (–0.51 to 1.12)
3 months	22	–0.13 (–0.71 to 0.45)	22	–0.17 (–0.75 to 0.40)	–0.04 (–0.86 to 0.78)
6 months	23	–0.12 (–0.70 to 0.46)	24	–0.04 (–0.60 to 0.53)	0.08 (–0.73 to 0.89)
1 year	23	–0.17 (–0.75 to 0.40)	24	–0.16 (–0.72 to 0.41)	0.02 (–0.79 to 0.82)
2 years	21	–0.58 (–1.17 to 0.01)	22	–0.11 (–0.69 to 0.47)	0.47 (–0.35 to 1.30)
5 years	18	–0.36 (–0.96 to 0.24)	20	–0.16 (–0.74 to 0.43)	0.20 (–0.64 to 1.04)
Z-rotation, degrees					
6 weeks	21	1.21 (0.25 to 2.17)	24	0.40 (–0.54 to 1.24)	–0.81 (–2.15 to 0.54)
3 months	22	1.26 (0.30 to 2.22)	22	0.70 (–0.25 to 1.64)	–0.57 (–1.91 to 0.78)
6 months	23	1.17 (0.21 to 2.13)	24	0.95 (0.01 to 1.88)	–0.22 (–1.57 to 1.12)
1 year	23	0.89 (–0.07 to 1.85)	24	1.04 (0.11 to 1.98)	0.16 (–1.19 to 1.50)
2 years	21	0.95 (–0.02 to 1.91)	22	0.94 (–0.01 to 1.88)	–0.01 (–1.36 to 1.34)
5 years	18	1.31 (0.33 to 2.28)	20	1.08 (0.12 to 2.03)	–0.23 (–1.60 to 1.13)
Total rotation, degrees					
6 weeks	21	2.46 (1.22 to 3.71)	24	1.90 (0.68 to 3.11)	–0.57 (–2.31 to 1.17)
3 months	22	3.02 (1.78 to 4.27)	22	2.26 (1.04 to 3.48)	–0.76 (–2.50 to 0.98)
6 months	23	3.34 (2.10 to 4.58)	24	2.26 (1.05 to 3.47)	–1.08 (–2.82 to 0.65)
1 year	23	3.06 (1.82 to 4.30)	24	2.22 (1.00 to 3.43)	–0.84 (–2.57 to 0.90)
2 years	21	2.99 (1.75 to 4.24)	22	2.31 (1.09 to 3.53)	–0.68 (–2.42 to 1.07)
5 years	18	2.75 (1.50 to 4.01)	20	2.07 (0.85 to 3.30)	–0.68 (–2.43 to 1.07)

### Translations

The monoblock CoC cup and the modular CoPE cup showed comparable translation patterns along the x-, y-, and z-axes during 5 years’ follow-up (see Table 4 and Figure 2). Both cups showed a statistically significant increase in translation along the x- and y-axes over time (P < 0.003), but statistically stabilized between 6 weeks and 3 months. The translations along the x- and y-axes of the monoblock CoC cup were larger than the precision interval at all time points, while only the translation along the y-axis of the modular CoPE cup was larger than the precision interval at all time points. When comparing the total translation, a significant difference between the cups was found at 5 years’ follow-up; the monoblock CoC cup showed a higher total translation (1.54 mm, CI 1.11–1.98) compared with the modular CoPE cup (0.84 mm, CI 0.42–1.26) (see Table 4 and Figure 4).

### Rotations

Both cups had comparable rotation patterns about the x-, y-, and z-axes during 5 years’ follow-up (see Table 5 and Figure 3). However, there was only a statistically significant change over time about the z-axis (P < 0.001), which statistically stabilized between 6 weeks and 3 months. Furthermore, only the rotation about the z-axis fell outside the precision interval of both cups, but not at all time points. Figure 3 shows that between postoperatively and 6 months an increase in rotation about the z-axis was found, after which the rotation decreased until 5 years after surgery. The total rotation shows the same pattern (see Figure 4). After 5 years, the cups showed a comparable total rotation; the monoblock CoC cup showed a mean total rotation of 2.75° (CI 1.50–4.01) and the modular CoPE cup a mean total rotation of 2.07° (CI 0.85–3.30) (Figure 4).

### PROMS

No difference occurred between the groups on responder/non-responder rates on MCII on the HHS after 5 years (69%, CI 42–88 in CoC monoblock cup vs 95%, CI 74–100 in CoPE modular cup, with OR 0.11, CI 0.01–1.06), on moderate improvement on the HHS after 2 years (41%, CI 22–63 vs 57%, CI 34–77, respectively, with OR 0.52, CI 0.16–1.75) and 5 years (19%, CI 5–46 vs 38%, CI 19–61, respectively, with OR 0.38, CI 0.08–1.74), on the OHS after 2 years (80%, CI 59–92 vs 83%, CI 61–94, respectively, with OR 0.84, CI 0.20–3.62), and on the OHS after 5 years (72%, CI 46–89 vs 90%, CI 66–98, respectively, with OR 0.31, CI 0.05–1.84) (Table 6). However, we found a difference between the groups on responder/non-responder rates on MCII on the HHS after 2 years (64%, CI 41–82 in CoC monoblock cup vs 95%, CI 74–100 in CoPE modular cups, with OR 0.09, CI 0.01–0.78) (Table 6). The EQ-5D scores are shown in Tables A1–A8 (see Supplementary data).

**Table 6:** HHS and OHS scores divided into responders and non-responders based on results at 2 and 5 years’ follow-up

Item	n	CoC MB	n	CoPE mod	Odds ratio (CI)
		n (%) [CI, %]		n (%) [CI, %]	
Oxford Hip Score, responders					
2 years	25	20 (80) [59–92]	23	19 (83) [61–94)	0.84 (0.20–3.62)
5 years	18	13 (72) [46–89]	19	17 (90) [66–98)	0.31 (0.05–1.84)
Harris Hip Score—MCII, responders					
2 years	22	14 (64) [41–82]	21	20 (95) [74–100)	0.09 (0.01–0.78)
5 years	16	11 (69) [42–88]	21	20 (95) [74–100)	0.11 (0.01–1.06)
Harris Hip Score—moderate improvement, responders					
2 years	22	9 (41) [22–63]	21	12 (57) [34–77)	0.52 (0.16–1.75)
5 years	16	3 (19) [5–46]	21	8 (38) [19–61)	0.38 (0.08–1.74)

MCII = minimal clinically important improvement.

CI = 95% confidence interval.

CoC MB = ceramic-on-ceramic monoblock.

CoPE mod = ceramic-on-polyethylene modular.

### Complications

No statistically significant difference in complication rates was found between the groups during 5 years of follow-up (Table 7). On all conventional radiographs, there were no signs of acetabular osteolysis, cup loosening or malpositioning. In the CoC monoblock cup group, 3 arthroplasties were complicated by intraoperative femoral fissures. Both groups had 1 patient each with a conservatively treated avulsion fracture of the greater trochanter after 6 weeks. In the CoPE modular cup group, 1 patient dislocated his hip twice and underwent closed reduction both times. 3 patients with unexplained persistent pain scored lower on functional outcome several times during follow-up. 1 patient in the CoPE modular cup group remained in pain 10 months after surgery. This cup was revised due to malpositioning of the cup. The patient was excluded from further follow-ups.

**Table 7:** Complications. Values are count and odds ratio (CI)

Item	CoC MB	CoPE mod	Odds ratio (CI)
	n = 25	n = 25	
Intraoperatively	3	–	–
Bone fissure in femur	3	–	
Postoperatively	3	6	0.43 (0.09–1.97)
Revision for loosening	–	1	
Avulsion fracture trochanter	1	1	
Collapse femoral stem	–	1	
Dislocation	–	1	
Persistent pain	1	2	
Death (not related to THA)	1	0	

For abbreviations, see Table 6.

## Discussion

This is the first study investigating migration and fixation patterns in an uncemented monoblock CoC acetabular cup and comparing it with an uncemented modular CoPE cup. The primary aim of this study was to determine fixation and migration patterns of the CoC monoblock cup, compared with the standardly used CoPE modular cup. Both cups show comparable migration patterns during 5 years’ follow-up and stabilized between 6 weeks and 3 months. However, the CoC monoblock cup shows a higher total translation compared with the CoPE modular cup, while rotations were similar for both cups. No differences were found in revision rates and clinical outcomes.

The higher total translation of the CoC monoblock cup can be explained by a higher, though not statistically significant, translation along the x-axis compared with the CoPE modular cup, while both cups translated comparably along the y-axis.

The proximal translation pattern of both cups is similar to that found by Cho et al., who studied the pooled proximal migration of several kinds of cup [27]. All cups showed an initial migration, after which the migration stabilized. However, compared with the pooled proximal migration of uncemented cups, namely 0.12 mm (CI 0.04–0.19), both the CoPE modular and CoC monoblock cup show a higher migration at 2 years’ follow-up. Klaassen et al. compared an uncemented CoC modular cup system with an uncemented CoPE cup [28]. They found comparable patterns, but, in contrast to our results, they did not find differences between the cups. Furthermore, we found a lower proximal translation and adduction (rotation about the z-axis) at 5 years’ follow-up in both cups compared with their results.

Proximal migration (translation along the y-axis) of the acetabular component has been found to be associated with the risk of revision on the long term. Pijls et al. described that a proximal migration of < 0.2 mm at 2 years of follow-up is associated with a risk of < 5% on revision due to aseptic loosening, while proximal migration of > 1.0 mm is associated with a risk of revision of > 10% due to aseptic loosening. The cups with a mean proximal migration between 0.2 and 1.0 mm were at > 5% risk of revision after 10 years [19]. In our study, the CoC monoblock cup showed a mean proximal migration of 0.50 mm at 2 years of follow-up, compared with 0.36 mm in the CoPE modular cup. According to Pijls et al., this means that both cups are at > 5% risk of revision at 10 years of follow-up, due to aseptic loosening. However, Pijls et al. mostly included studies that analyzed cemented cups, which might diminish applicability to the cups in our study.

One advantage of the CoC monoblock cup was the provision of large femoral heads. In past studies, total hip arthroplasty with large femoral heads has been shown not to increase revision rates due to aseptic loosening [29,30]. In our study, the CoC monoblock cup contains larger femoral heads than the CoPE modular cup. The results in our study are comparable to previous meta-analyses [29,30].

### Strengths

This is an RCT comparing 2 uncemented acetabular components. All arthroplasties within this study were performed with the lateral approach by one orthopedic surgeon. We use RSA to quantify migration patterns, thereby contributing to the body of knowledge on migration patterns of different prosthesis designs.

### Limitations

We did not achieve 100% follow-up on RSA at all follow-up moments and on PROMs, partly due to the COVID-19 pandemic.

### Conclusion

We showed that the uncemented CoC monoblock acetabular cup system showed comparable migration patterns but had a higher translation than the uncemented modular CoPE acetabular cup system. This migration does not increase the risk of revision according to established migration thresholds. Mid-term migration rates and clinical outcomes were similar after 5 years of follow-up.

### Supplementary data

Tables A1–A8 are available as Supplementary data on the article home page, doi: 10.2340/17453674.2026.46138

## Supplementary Material



## Notes

RMB and NMCM designed the study. RMB informed patients and performed the surgeries. RMB followed up the patients. IB took the radiographs. JHP and IB analyzed the radiographs. JHP, BH, and CI analyzed the data and wrote the manuscript. All authors critically reviewed the manuscript and approved the manuscript for submission.

The authors would like to thank Nicole de Esch, research nurse, for informing and including patients and coordinating follow-up visits. Also, they would like to thank Rianne Oomen, research coordinator, for analyzing the radiographs.

Handling co-editors: Per Henrik Randsborg and Robin Christensen

*Acta* thanks Eduardo García-Rey and Ian Harris for help with peer review of this manuscript.
