# Supplementary material for: Migration in large femoral head ceramic-on-ceramic monoblock acetabular component compared with modular ceramic-on-polyethylene acetabular component in total hip arthroplasty using radiostereometric analysis with 5 years’ follow-up: a randomized controlled trial
Source: Acta Orthop. 2026 Jul 1;97:448–55. doi: 10.2340/17453674.2026.46138 (PMC13320593; doi:10.2340/17453674.2026.46138)
Supplement: Supplementary file 1 [file ActaO-97-46138-s1.pdf]

Supplementary data

Table A1. EQ-5D VAS scores. Values are mean (standard deviation) [95% confidence interval]

	Maxera cup (n = 25)	Allofit cup (n = 25)
Baseline	69.2 (18.1) [61.7–76.7]	69.5 (18.0) [61.7–77.2]
6 weeks	75.0 (15.4) [68.6–81.3]	72.8 (18.3) [65.1–80.5]
3 months	79.6 (13.3) [73.9–85.4]	73.5 (16.6) [66.5–80.5]
6 months	76.0 (19.8) [67.8–84.1]	73.1 (17.6) [65.7–80.6]
1 year	77.9 (17.1) [70.7–85.1]	79.1 (16.1) [71.9–86.2]
2 years	76.6 (16.8) [69.6–83.5]	80.5 (16.7) [73.5–87.6]
5 years	77.2 (17.6) [68.2–86.3]	85.2 (16.5) [77.7–92.7]

Table A2. Baseline EQ-5D-scores. Values are count

	Maxera cup (n = 25)	Allofit cup (n = 23)
Mobility		
No problems	–	1
Slight problems	2	2
Moderate problems	13	9
Severe problems	10	11
Unable	–	–
Self-care		
No problems	17	8
Slight problems	2	6
Moderate problems	6	9
Severe problems	–	–
Unable	–	–
Usual activities		
No problems	3	1
Slight problems	5	6
Moderate problems	11	9
Severe problems	6	5
Unable	–	2
Pain / discomfort		
None	1	–
Slight	3	1
Moderate	11	12
Severe	10	10
Extreme	–	–
Anxiety/depression		
None	16	20
Slight	7	2
Moderate	1	1
Severe	1	–
Extreme	–	–

Table A3. EQ-5D-scores at 6 weeks. Values are count

	Maxera cup (n = 25)	Allofit cup (n = 24)
Mobility		
No problems	2	1
Slight problems	13	10
Moderate problems	8	11
Severe problems	2	2
Unable	–	–
Self-care		
No problems	12	12
Slight problems	10	6
Moderate problems	3	5
Severe problems	–	1
Unable	–	–
Usual activities		
No problems	6	4
Slight problems	10	7
Moderate problems	9	11
Severe problems	–	2
Unable	–	–
Pain / discomfort		
None	3	1
Slight	15	13
Moderate	7	9
Severe	–	1
Extreme	–	–
Anxiety/depression		
None	22	17
Slight	3	4
Moderate	–	2
Severe	–	1
Extreme	–	–

Table A4. EQ-5D-scores at 3 months. Values are count

	Maxera cup (n = 23)	Allofit cup (n = 24)
Mobility		
No problems	2	4
Slight problems	16	12
Moderate problems	4	8
Severe problems	1	–
Unable	–	–
Self-care		
No problems	20	18
Slight problems	3	6
Moderate problems	–	–
Severe problems	–	–
Unable	–	–
Usual activities		
No problems	9	7
Slight problems	9	12
Moderate problems	5	5
Severe problems	–	–
Unable	–	–
Pain / discomfort		
None	3	5
Slight	15	16
Moderate	4	3
Severe	1	–
Extreme	–	–
Anxiety/depression		
None	21	19
Slight	2	3
Moderate	–	2
Severe	–	–
Extreme	–	–

Table A5. EQ-5D-scores at 6 months. Values are count

	Maxera cup (n = 23)	Allofit cup (n = 24)
Mobility		
No problems	2	4
Slight problems	16	12
Moderate problems	4	8
Severe problems	1	–
Unable	–	–
Self-care		
No problems	20	18
Slight problems	3	6
Moderate problems	–	–
Severe problems	–	–
Unable	–	–
Usual activities		
No problems	9	7
Slight problems	9	12
Moderate problems	5	5
Severe problems	–	–
Unable	–	–-
Pain / discomfort		
None	3	5
Slight	15	16
Moderate	4	3
Severe	1	–
Extreme	–	–
Anxiety/depression		
None	21	19
Slight	2	3
Moderate	–	2
Severe	–	–
Extreme	–	–

Table A6. EQ-5D-scores at 1 year. Values are count

	Maxera cup (n = 25)	Allofit cup (n = 22)
Mobility		
No problems	8	10
Slight problems	11	7
Moderate problems	4	3
Severe problems	2	2
Unable	–	–
Self-care		
No problems	17	17
Slight problems	7	4
Moderate problems	1	1
Severe problems	–	–
Unable	–	–
Usual activities		
No problems	14	11
Slight problems	7	8
Moderate problems	3	3
Severe problems	1	–
Unable	–	–
Pain / discomfort		
None	11	8
Slight	10	8
Moderate	2	3
Severe	2	2
Extreme	–	1
Anxiety/depression		
None	23	18
Slight	2	3
Moderate	–	1
Severe	–	–
Extreme	–	–

Table A7. EQ-5D-scores at 2 years. Values are count

	Maxera cup (n = 25)	Allofit cup (n = 24)
Mobility		
No problems	11	13
Slight problems	9	5
Moderate problems	4	4
Severe problems	1	2
Unable	–	–
Self-care		
No problems	20	20
Slight problems	4	2
Moderate problems	1	2
Severe problems	–	–
Unable	–	–
Usual activities		
No problems	14	14
Slight problems	8	5
Moderate problems	2	5
Severe problems	1	–
Unable	–	–
Pain / discomfort		
None	9	13
Slight	10	6
Moderate	5	5
Severe	1	–
Extreme	–	–
Anxiety/depression		
None	24	20
Slight	1	3
Moderate	–	1
Severe	–	–
Extreme	–	–-

Table A8. EQ-5D-scores at 5 years. Values are count

	Maxera cup (n = 17)	Allofit cup (n = 21)
Mobility		
No problems	5	13
Slight problems	10	5
Moderate problems	1	2
Severe problems	1	1
Unable	–	–
Self-care		
No problems	14	19
Slight problems	3	2
Moderate problems	–	–
Severe problems	–	–
Unable	–	–
Usual activities		
No problems	10	17
Slight problems	5	2
Moderate problems	1	2
Severe problems	1	–
Unable	–	–
Pain / discomfort		
None	9	12
Slight	6	7
Moderate	–	1
Severe	2	1
Extreme	–	–
Anxiety/depression		
None	15	20
Slight	1	–
Moderate	1	–
Severe	–	1
Extreme	–	–
